# Urgency as an Endpoint in Irritable Bowel Syndrome

**DOI:** 10.4021/gr283e

**Published:** 2011-01-20

**Authors:** Allen W. Mangel, Jianmin Wang, Beth Sherrill, Ari Gnanasakthy, Claire Ervin, Sheri E. Fehnel

**Affiliations:** a RTI Health Solutions, Research Triangle Park, NC, USA; b Novartis Pharmaceuticals, East Hanover, NJ, USA

**Keywords:** Diarrhea-predominant irritable bowel syndrome, Urgency, Stool frequency, Stool consistency, Patient-reported outcomes

## Abstract

**Background:**

The choice of endpoints is crucial for proper evaluation of agents in clinical trials of irritable bowel syndrome (IBS). In a recently published draft guidance for IBS from the United States Food and Drug Administration (FDA), urgency was not considered an appropriate primary endpoint. The FDA’s position is that it is not clear how patients with diarrhea-predominant IBS (D-IBS) “define or describe urgency”. The aims of this study were to evaluate the association of urgency with stool frequency and consistency in patients with D-IBS and to describe results from patient interviews on their understanding of the term urgency.

**Methods:**

A retrospective analysis of clinical trial data in patients with D-IBS was conducted. Analyses focused on the relationship of urgency to stool frequency and consistency. Interviews were conducted with patients with D-IBS to test their understanding of the term urgency.

**Results:**

On the days that patients reported urgency, as compared to the days that patients did not report urgency, they had more frequent bowel movements (3.9 versus 1.8) and looser stools (Bristol Stool Score: 5.4 versus 4.2). The differences for both parameters, evaluated on the days with or without urgency, were statistically significant. In patient interviews, patients with D-IBS had a clear understanding of the concept and terminology of urgency and considered it one of their two most bothersome symptoms.

**Conclusions:**

Urgency should be considered a suitable co-primary endpoint in D-IBS studies.

## Introduction

The selection and use of patient-reported outcome (PRO) measures in clinical trials of irritable bowel syndrome (IBS) has been an active area of discussion over the past several years. In 2010, the United States Food and Drug Administration (FDA) published a draft guidance on endpoints in IBS trials [1]. In that document, the position is stated that urgency is not a concept that patients can clearly interpret, and therefore, it should not be considered an appropriate primary or co-primary endpoint for clinical trials. “It is not clear how patients define or describe urgency and what terminology will appropriately capture this symptom from the patient’s perspective.” [1]

IBS-related urgency (urgency, hereafter) like pain, diarrhea, constipation, and other symptoms, carries no formal definition. However, urgency is generally considered the unpleasant sensation that one needs to rush to the toilet or they may soil themselves. Although urgency is most common in patients with diarrhea-predominant IBS (D-IBS), patients with constipation-predominant IBS and alternating IBS also report urgency [2].

As part of two large, double-blind, placebo-controlled D-IBS clinical trials, a survey was conducted in which patients were asked, “When your IBS is active, which of the following is your most bothersome symptom?” [3] The survey was conducted twice, and in each instance, urgency was considered the second most bothersome symptom, second only to pain [3].

The therapeutic goal of D-IBS treatments is to improve pain and abnormal bowel function. Some D-IBS drugs improve stool frequency and consistency, but overshooting a “normal” bowel state and the development of constipation is an issue [4, 5]. Constipation is a potentially serious medical problem whether occurring spontaneously or drug-induced [6, 7].

IBS clinical trial data are currently evaluated as representing greater therapeutic benefit when symptom change from baseline is larger. This paradigm can lead to misinterpretation of frequency and consistency patterns because there is a U-shaped continuum of ideal stool movement. In other words, the direction of improvement for frequency and consistency symptoms is ambiguous, depending on the starting state (diarrhea or constipation). Furthermore, simple change from baseline cannot distinguish between a treatment that normalizes bowel function and one that induces the opposite extreme. For example, a patient with D-IBS is considered improved if stool frequency decreases and stool consistency hardens, even if the patient moves from a state of diarrhea to normal and then to constipation. This is a critical concept to consider because the therapeutic goal is not to make patients with D-IBS constipated or to induce diarrhea in patients with constipation-predominant IBS. By contrast, for pain and urgency, directionality of improvement is unambiguous; a reduction is always beneficial.

This study evaluated the association between stool frequency and consistency with urgency in patients with D-IBS. Qualitative interview data demonstrating patients’ understanding of urgency are also provided. We believe these data provide important new information that can contribute to better measurement of drug efficacy in IBS.

## Methods

### Patient symptom data

The data analyzed came from the 2-week screening period of a randomized, double-blind, multicenter, phase 2 IBS study that was conducted in 120 sites located in the United States. The trial evaluated the efficacy and safety of a novel therapeutic agent and placebo (Clinical Trials.gov identifier NCT00454688) [2]. The study was conducted under approval from an institutional review board, and patients signed informed consent before screening. Patients could be male or female with at least a 6-month history of IBS symptoms who met the Rome II criteria for IBS [8]. Patients had to fulfill entry pain criteria, at least halfway between mild and moderate pain; have at least one bowel movement during each of the 2 screening weeks; and be compliant with the data collection system (interactive voice response system). Exclusionary conditions included histories of drug or alcohol abuse, suicide attempt, hospitalization for a major psychiatric disorder within the past 2 years, pregnancy, exclusionary laboratory values, and various medical conditions, surgeries, and medications.

The study enrolled all subtypes of IBS but only patients with D-IBS were used for these analyses. Patients were categorized as D-IBS if diarrhea was their main bowel disturbance at least 75% of the time when their IBS was active. Data were analyzed from the 2-week pretreatment screening phase during which patients recorded daily self-assessments including, but not limited to, the presence or absence of urgency, number of bowel movements passed that calendar day, and average stool consistency using the Bristol Scale [9]. Descriptive statistics, such as means and percentages, were used to depict the patient characteristics observed at the start of screening. A linear mixed model was used to assess the association of urgency with frequency, incorporating repeated measurements across all screening days and separate per-patient intercepts; this analysis was repeated for the relationship between urgency and daily consistency scores.

### Patient interviews

Two iterative sets of cognitive debriefing interviews were conducted with a total of 20 patients with D-IBS (10 per set) to pretest and refine a series of PRO items before their use in D-IBS clinical trials. There was no overlap between the clinical trial population and the patients interviewed. Interview participants were recruited by the two focus group facilities hosting the interviews with assistance from a local gastroenterologist as necessary. To be eligible for interview participation, patients were required to be female; be at least 18 years old; have an existing diagnosis of IBS; have a presence of IBS symptoms within the past 3 months; and report diarrhea as their primary bowel symptom when their IBS was active.

Two female interviewers conducted all 20 patient interviews using a semistructured interview guide. At the start of each interview, participants were asked a series of general, open-ended questions designed to get them talking about their IBS symptoms and related experiences. The discussion then transitioned into cognitive debriefing of the draft PRO items. Specifically, a “think-aloud” technique was used, wherein the interviewers asked the participants to read the instructions and questions aloud and describe, in their own words, how they interpreted each item. Follow-up probes were also posed by the interviewers to further elucidate the comprehension and response process. In the second set of interviews, participants were also asked to rank order a series of six IBS symptoms commonly reported by participants in the first set of interviews (urgency, abdominal pain, abdominal discomfort, frequent bowel movements, bloating, and loose or watery stools) by degree of bothersomeness. The results of the ranking task were scored such that each symptom was assigned a number from 1 to 6 with 1 being the most bothersome and 6 being the least bothersome. Although a wide variety of PRO items were tested, only results pertaining to the item addressing urgency are relevant to this paper and are, therefore, reported below.

## Results

### Daily stool frequency and consistency on days with and without urgency

One hundred ninety-three patients with D-IBS were included in this analysis, 69% (n = 134) were female and 92% (n = 177) were Caucasian. Patients had an average (± SEM) age of 48.6 ± 1.0 years. This demographic profile of a female, Caucasian patient in the age range of 40 - 50 years is prototypical for patients with IBS.

Assessments during the screening period were available for 2,845 days for the 193 patients; on 80% of these days, patients reported urgency. Based on the repeated measurement model, days with and without urgency corresponded to stool frequencies of 3.87± 0.17 (mean ± SEM) versus 2.34 ± 0.20, respectively, with a difference of 1.52 ± 0.13, %95 CI (1.28 - 1.77) and P value < 0.0001. Similarly, examination of consistency values using the Bristol Stool Score on days with and without urgency revealed scores of 5.31 ± 0.06 versus 4.23 ± 0.09, respectively, with difference of 1.08 ± 0.07, %95 CI (0.93 - 1.22) and P value < 0.0001. This 1-point average difference in the Bristol scale refers to smooth, soft bowel movements like sausage to soft blobs with clear-cut edges [9]. We consider the difference in stool frequency and consistency for days with and without urgency as highly clinically relevant, comparable to results with D-IBS medications that show similar average changes in daily bowel frequency and consistency [4, 5].

### D-IBS patient interviews

Ten female patients participated in each of two iterative sets of cognitive debriefing interviews. Patients had an average (± SEM) age of 53.2 ± 2.4 in Group 1 and an average age of 39.7 ± 4.0 years in Group 2. Seventy percent of patients in Group 1 were Caucasian and 80% in Group 2. All patients in each group reported being diagnosed with IBS by a physician before screening (range of time since diagnosis - Group 1: 2 - 40 years; Group 2: 10 months to 10 years).

When asked to describe their experiences in relation to IBS, participants in Group 1 mentioned a variety of physical symptoms, including urgency, abdominal pain, cramping, bloating, nausea, diarrhea, frequent bowel movements, and gas. Many participants described episodes of fecal incontinence and feelings they subsequently associated with urgency, including anxiety, fear, and worry. The most bothersome symptoms reported by participants in Group 1 generally included urgency, abdominal pain or cramping, and diarrhea.

Three dichotomous (yes/no) items addressing the experience of urgency were debriefed with the Group 1 participants: Q1: Did you experience bowel urgency today? Q2: Did you experience bowel urgency today? (Bowel urgency means that when you feel the need for a bowel movement, you have to rush to the toilet to avoid an accident.) Q3: Did you experience urgency for bowel movement today? (Urgency for bowel movement means that when you feel the strong need to have a bowel movement, you have to rush to the toilet to avoid an accident.)

Interview participants consistently indicated that question 1 would be easy for them to answer on a daily basis, describing “urgency” as “have to go immediately” or “can’t hold it”. Despite the ease with which the concept of urgency was understood by the interview participants, these patients generally preferred question 2 to question 1, indicating that a definition could be helpful to others completing the question in the future and that the definition provided was appropriate. On the other hand, the wording in question 3 (“urgency for bowel movement”) was viewed as awkward by the interview participants; 8 of 10 Group 1 patients indicated a definite preference for question 2 over question 3. In summary, although all participants articulated similar definitions for bowel urgency, they suggested that providing a definition for this concept in future studies could be helpful to patients and preferred the wording in question 2.

In the second set of interviews, only question 2 was evaluated because it was the strong preference of patients in Group 1. In Group 2, 10 out of 10 patients reported that question 2 was clear and easy to answer. Group 2 participants were asked to rank order six IBS symptoms commonly reported by Group 1 participants; average scores for urgency and abdominal pain were the most bothersome with scores of 2.1 and 2.2, respectively. The order of the next most bothersome symptoms and their respective scores were frequent bowel movements, 3.2; abdominal bloating, 4.0; loose or watery stools, 4.2; and abdominal discomfort, 4.7.

## Discussion

In disorders such as IBS where no pathognomonic laboratory, radiographic, endoscopic, or pathologic markers exist either for the initial disease diagnosis or to follow the progression of the disease, PROs represent the marker for assessing improvement during a clinical trial. IBS is characterized by abdominal pain with altered bowel functions. Standard measures of bowel activity include scoring the number of bowel movements in a day and measuring stool consistency. A flaw with either stool frequency or consistency measurements in clinical trials is that as patients move from one state (i.e., diarrhea or constipation) through a state of normal bowel habits to the opposite state (e.g., diarrhea to constipation) the standard instruments are analyzed to record these changes as greater therapeutic benefit.

By contrast, the directional interpretation of urgency, like pain, is clear. Urgency and pain cannot overshoot as stool frequency and stool consistency can, and urgency, therefore, does not have the potential to show this “false therapeutic benefit” if a patient with D-IBS becomes constipated. As reported in the patient interviews, as well as in a previous study [3], urgency is a clinically important endpoint and was as bothersome to patients as pain. The fear of soiling one’s undergarments makes patients much more likely to be confined to home and not travel far away from a known location where there is a toilet [10].

In the present study, we provide support that urgency is an understandable concept to patients. First, days with urgency were significantly associated with both increased frequency of daily stools and looser consistency scores. These associations were consistent with intuitive definitions of urgency, as well as those indicated by patients. Furthermore, as demonstrated in the patient interviews, urgency was a readily understandable concept by patients with D-IBS. The data in this study were collected from patients in the United Stated and cultural translations of the term urgency merit inspection.

There remains a consequence of regulatory agencies not accepting urgency as a primary endpoint in D-IBS clinical trials: drugs that make patients with D-IBS constipated will appear to have greater efficacy than agents that take patients from a diarrhea-like state to a more normal state. Development of agents that normalize, rather than constipate, bowel states in patients with D-IBS should be encouraged because the clinical consequences of drug-induced constipation can span the spectrum of annoying to life-threatening [6, 7]. Thus, we suggest that urgency is both understandable and clinically meaningful to patients with D-IBS and should represent an acceptable co-primary endpoint with pain in D-IBS clinical trials.
